# Generative Large Model-Driven Methodology for Color Matching and Shape Design in IP Products

**DOI:** 10.3390/e27030319

**Published:** 2025-03-19

**Authors:** Fan Wu, Peng Lu, Shih-Wen Hsiao

**Affiliations:** 1 Department of Product Design, Dalian Polytechnic University, Dalian 116034, China; wuxiaofan999@gmail.com; 2 Department of Industrial Design, Dalian University of Technology, Dalian 116024, China; 3 Department of Industrial Design, National Cheng Kung University, Tainan 70101, Taiwan; swhsiao@mail.ncku.edu.tw

**Keywords:** product design, shape design, color matching, quadratic curvature entropy, tourism IP product

## Abstract

The rise in generative large models has gradually influenced traditional product design processes, with AI-generated content (AIGC) playing an increasingly significant role. Globally, tourism IP cultural products are crucial for promoting sustainable tourism development. However, there is a lack of practical design methodologies incorporating generative large models for tourism IP cultural products. Therefore, this study proposes a methodology for the color matching and shape design of tourism IP cultural products using multimodal generative large models. The process includes four phases, as follows: (1) GPT-4o is used to explore visitors’ emotional needs and identify target imagery; (2) Midjourney generates shape options that align with the target imagery, and the optimal shape is selected through quadratic curvature entropy method based on shape curves; (3) Midjourney generates colored images reflecting the target imagery, and representative colors are selected using AHP and OpenCV; and (4) color harmony calculations are used to identify the best color combination. These alternatives are evaluated quantitatively and qualitatively using a color-matching aesthetic measurement formula and a sensibility questionnaire. The effectiveness of the methodology is demonstrated through a case study on the harbor seal, showing a strong correlation between quantitative and qualitative evaluations, confirming its effectiveness in tourism IP product design.

## 1. Introduction

With the advancement of intelligent manufacturing technologies, products have become increasingly homogeneous in terms of processes, functions, and performance, while exhibiting greater diversity in visual aesthetic features such as color matching, shape, material, texture, and pattern [1,2,3]. Many scholars argue that products with visual aesthetics that align with consumers’ emotional needs are more likely to attract attention and stimulate purchasing desire, ultimately boosting sales [4,5,6]. Therefore, regardless of the product type, visual aesthetic features should be given significant consideration during the product development phase. Among various visual aesthetic features, shape and color matching are the two core design characteristics in product development [3,7]. For years, both academia and industry have commonly employed Kansei engineering to establish models that link consumers’ emotional needs with product design features, aiming to translate abstract emotional demands into tangible product shapes and color-matching schemes [8,9].

Regarding Kansei design for product shape, Lo et al. [10] used six form aesthetic-related Kansei words as evaluation indicators and combined genetic algorithms with fuzzy theory to create a shape design and evaluation method. Liu et al. [11] proposed a methodology for the shape design of cultural and creative products based on Kansei engineering, factor analysis, and triangular fuzzy numbers. Zhou et al. [12] introduced a car frontal form design and evaluation method using Kansei engineering and convolutional neural networks. Recently, Wu et al. [13] developed an intelligent design system that uses generative adversarial networks to transform product hand-drawn sketches into designs that match the target imagery. Yuan et al. [6] established an aesthetic measurement evaluation index consisting of eight form aesthetics Kansei words, and combined Kansei engineering, genetic algorithms, and eye-tracking technology to propose a design method for automotive forms. Lu et al. [14] introduced an improved form aesthetic quantification formula and combined it with Kansei engineering and the finite structure method to propose a product shape design and evaluation method.

In addition, regarding Kansei design for product color matching, Tsai and Chou [15] proposed a color-matching design method for two-color combination products based on Kansei engineering, color harmony, color association, and genetic algorithms. Hsiao et al. [16] developed a model for quantifying product color-matching aesthetics based on parameters such as hue, value, chroma, and the area of colored regions. This model addressed imagery adjective-guided product color-matching design and was applied to the Kansei design of products [17]. Moreover, Hsiao and Tsai [18] suggested that the color imageries found in colored images (e.g., natural images and works of famous painters) could be transferred to product color matching in Kansei design. They developed a color-matching design system based on Kansei engineering and fuzzy theory, demonstrating its feasibility. Recently, Lu and Hsiao [3] expanded the feasibility of the product color-matching aesthetics quantification model and Kansei design by considering the impact of different observation angles on product color matching. Wu et al. [7] proposed three methodologies to extract colors from natural images for product color matching, utilizing Kansei engineering, AIGC, and color harmony theory. Table 1 presents a concise comparison of the key characteristics of these studies. In these studies, combining Kansei engineering with product color matching and shape design, imagery adjectives are commonly used as a medium to build models linking consumer emotional needs with product design features.

In recent years, with the development of artificial intelligence, and big data processing, scholars have constructed generative models to address various specific design problems using machine learning techniques like genetic algorithms and neural networks [13,19,20]. Recently, many emerging tech companies have developed various generative large models based on deep learning networks, such as CNNs, GANs, diffusion models, and transformer models. Specifically, generative large models can be categorized based on different types of generated content (i.e., artificial intelligence generated content, AIGC), including text, image, audio, and video generative models [7]. Currently, the most popular text generative large model globally is ChatGPT (https://chatgpt.com/), with its latest version updated to GPT-4.5 as of March 2025. Due to its use of large language models (LLMs) and advanced natural language processing techniques during training, ChatGPT is capable of handling complex text generation tasks such as writing, translation, programming, and text analysis [21,22].

In addition, Midjourney, Stable Diffusion, and DALL-E are considered the leading image generative large models internationally [23,24,25]. These models are trained using millions of text-to-image examples and complex deep learning networks, enabling them to generate creative and high-quality images. Since the emergence of generative large models, many scholars have actively applied them to product design. Lu et al. [9] proposed an automotive shape design method based on ChatGPT, Midjourney, and Stable Diffusion, demonstrating the feasibility of collaboration between generative large models and Kansei engineering. The results showed that generative large models can not only understand abstract user needs but also translate imagery adjectives into concrete product shapes. Wu et al. [7] proposed several color-matching design methods for household vacuum cleaners based on ChatGPT and Midjourney. The findings revealed that Midjourney could generate colored images that align with the imagery adjectives, providing effective color combinations for target imagery-oriented color design. Du et al. [26] introduced a Kansei design approach for product shape design based on the 2D sketch-to-3D rendering capability of Stable Diffusion, illustrating the effectiveness of the LoRA model (i.e., stylization model) in optimizing design efficiency. Wang et al. [27] used Midjourney to generate a large number of forms for reference in designing female electric scooter shapes, enhancing the efficiency of product shape design. Wang et al. [28] explored the feasibility of applying Midjourney to Ming Dynasty furniture design, paving a new path for the innovative design of furniture shapes. These studies have demonstrated that generative large models positively contribute to product color matching and shape design, optimizing traditional design processes and improving design efficiency.

Based on the aforementioned research background, the motivations for this study are as follows: (1) Although many scholars recognize that color matching [6,10,11,12,13,14] and shape [15,16,17,18] are the two core design features in product development, and various methodologies for color matching or shape design have been proposed based on Kansei engineering, few have addressed both target imagery-oriented color matching and shape design simultaneously [3,7]. Therefore, achieving consistency between color matching and shape toward the same target imagery during product development can significantly enhance the efficiency of Kansei design. (2) Recently, although several scholars have demonstrated the contribution of generative large models to improving product design efficiency through case studies [26,27,28], there is still a lack of examples showcasing collaboration among multiple generative large models. Therefore, presenting a case involving the collaboration of multiple generative large models would help inspire product designers to explore more flexible uses of generative models. (3) In the existing research, whether it is color matching design [7] or shape design [9,26,27,28] based on generative large models, the importance of evaluating the aesthetics of generated content has not been emphasized, nor have practical quantitative methods for aesthetic evaluation been provided to select the optimal shape or color combinations. Therefore, incorporating scientifically valid quantitative aesthetic evaluation methods would enhance the rigor and usability of design methodologies.

In summary, this study integrates the text-generative large model ChatGPT, the image-generative large model Midjourney, color harmony theory, and quantitative evaluation of shape and color matching to propose a design methodology that considers both product color and shape. Given the importance of tourism IP cultural products in promoting sustainable tourism development, this study uses the color matching and shape design of the Harbor Seal IP cultural product in Dalian as a case study. The structure of this paper is as follows: Section 2 introduces the theories and research methods used in this study. Section 3 presents the implementation steps of the generative large model-driven methodology. In Section 4, the Harbor Seal IP cultural product from Dalian is used as an example to demonstrate the proposed design methodology in detail and confirm its effectiveness. Section 5 discusses the research results and analyzes this study’s limitations. Finally, the conclusion highlights the theoretical and practical contributions made by this study.

## 2. Related Theories and Methods

### 2.1. Generative Large Models

Generative large models (GLMs) refer to advanced artificial intelligence systems that achieve automatic content generation through deep learning of large-scale datasets [29]. GLMs have become one of the frontiers of artificial intelligence technology, capable of generating text, images, audio, and video as AIGC based on user-provided prompts [30]. The core technologies of GLMs include diffusion models, generative adversarial networks (GANs), and the self-attention mechanism [31], which ensure the accuracy and practicality of generated content. Compared with traditional machine learning models, GLMs possess a strong ability to understand context and create content, generating multimodal outputs that align with semantics, logic, and user expectations based on input information. Currently, GLMs are widely applied across various fields, driving innovation and advancement in numerous industries. Internationally, ChatGPT is recognized as the most well-known text generative large model, while Midjourney is one of the most notable image generative large models.

#### 2.1.1. ChatGPT

ChatGPT is a text generative large model (text-GLM) created by OpenAI utilizing the generative pre-trained transformer (GPT). It can automatically produce fluent sentences based on written prompts [21]. Since its initial release in November 2022, versions have evolved from ChatGPT 3.5 to GPT-4.0, and subsequently to GPT-4o. Over the past few years, OpenAI has continuously incorporated the latest training data and applied advanced algorithms to train and update ChatGPT, making it a novel productivity tool for writing, literature analysis, programming, translation, and brainstorming [22]. To enable multimodal generation within a single interface, OpenAI integrated the self-developed image generative large model DALL-E into ChatGPT, allowing the newer versions (i.e., GPT-4.0 and GPT-4o) to generate both text and images. Moreover, GPT-4.0 and GPT-4o support various practical plugins, including the WebPilot extension, which enables real-time access to internet web pages and content analysis. In addition, to better guide ChatGPT in generating expected output, users are encouraged to design and optimize input prompts—a process known as prompt engineering [32]. Specifically, a ChatGPT prompt typically consists of several key components, including context, instruction, output indicator, and input data [9]. For instance, “We are working on a tourism IP cultural product for Dalian (context). Please provide some design suggestions based on visitor preferences (instruction), within 300 words (output indicator), focusing on aspects such as color matching, shape, and material (input data)”. Additionally, to fully leverage ChatGPT’s potential, researchers and enthusiasts have discovered useful prompt techniques, such as asking ChatGPT to respond in the role of a senior professional (e.g., “Please play a senior [role]”) or to generate an introduction on a specific topic (e.g., “Write an introduction to [topic]”).

In the context of product design, with the capabilities of prompt engineering, large language models, and practical extension plugins, ChatGPT has demonstrated significant application potential, including user needs research, product status analysis, design suggestions, and brainstorming [7]. Moreover, based on the self-attention mechanism and transformer architecture, ChatGPT can quickly understand and learn the context of prompts, thereby generating the expected output. In other words, ChatGPT can precisely learn from user-provided prompt examples, enabling it to serve as a prompt creator for other generative large models. In this study, ChatGPT is utilized in the preparation phase of the proposed design methodology, involving user needs research, internet information analysis, and prompt generation.

#### 2.1.2. Midjourney

Midjourney is an image generative large model (image-GLM) developed by Midjourney Technologies, capable of generating high-quality image content based on user-provided text prompts [23]. Since its release in February 2022, Midjourney has launched new versions every few months, progressing from version V1 to the current V6.2 version as of November 2024. With each version update, Midjourney has continually integrated cutting-edge algorithms, including CNNs, GANs, diffusion models, and transformer models, enhancing the quality, precision, accuracy, and creativity of its generated images. Compared to other image generative models like Stable Diffusion and DALL-E, Midjourney places greater emphasis on image quality, detail processing, and the expression of artistic style and emotion [27]. Research has demonstrated that Midjourney leverages large language models to produce high-quality images from concise prompts, effectively converting abstract subphrases (e.g., Kansei words, imagery adjectives, and art styles) into concrete visual content [7,9,24]. In addition, to ensure that the generated images meet user expectations, proficiency in prompt engineering is essential. Specifically, Midjourney prompts consist of various prompt subphrases, such as {target object, imagery style, scene effect, background settings, image resolution, and additional control parameters}. Moreover, Midjourney includes multiple useful commands, such as “/imagine”, “/blend”, and “/describe”, which support text-to-image, image-to-image, and image-to-text modes, respectively. Besides commands and modes, Midjourney offers various control parameters, with detailed descriptions provided in the literature [7].

In terms of product design applications, studies [7,9,24] have demonstrated that Midjourney can transform users’ emotional needs into concrete product shapes and color matching, opening new pathways for Kansei design. In this paper, Midjourney is employed in the shape generation and color generation phases of the proposed design methodology. Specifically, target imageries can be used as key prompt subphrases in Midjourney to generate corresponding product shapes and colored images.

### 2.2. Quantitative Calculation of Color Matching

#### 2.2.1. Color Harmony

Birkhoff [33] proposed that vague, uncertain, and subjective aesthetic perceptions can be quantified using mathematical models and introduced the first mathematical model for aesthetic measurement, expressed as *M* = *O*/*C*, where *M*, *O*, and *C* represent the aesthetic measure, order, and complexity of the evaluated object, respectively. Building on this foundation, Moon and Spencer [34,35,36] introduced the concept of color harmony in 1944, defining it as a combination of colors that through orderly variation, evokes a pleasing experience. To quantitatively assess the aesthetic measurement (*M*) of a color harmony, Moon and Spencer proposed a model using the Munsell Color System of *M* = *O*/*C*. In this model, *M* indicates the aesthetic measurement, *O* denotes the order, and *C* refers to the complexity of the color combination. The complexity (*C*) is determined by the following four parameters: the total number of colors ($N_{c}$), the logarithmic difference in hues ($N_{hd}$), values ($N_{vd}$), and chromas ($N_{cd}$), as shown in Equation (1).

(1) $$C=N_{c}+N_{hd}+N_{vd}+N_{cd}$$

Additionally, Moon and Spencer devised a paired comparison approach using the Munsell color system to determine the order (*O*) of a color combination. In this method, any two colors in a combination can have a relationship defined in terms of hue, value, and chroma as either a comfortable interval or an uncomfortable interval. Specifically, comfortable intervals are further categorized into identity, similarity, and contrast, whereas uncomfortable intervals are classified as first ambiguity, second ambiguity, and glare. Table 2 provides the quantitative ranges and corresponding scores for the six types of relationships involving hue, value, and chroma. Moreover, the order (*O*) of a color combination considers not only the differences in hue, value, and chroma between paired colors but also the effect value of area balance. Based on these parameters, the order (*O*) of a color combination can be expressed as in Equation (2) [3].

(2) $$O={\alpha h}_{i}+{\alpha v}_{i}+{\alpha c}_{i}+A_{b_{i}}$$

where ${\alpha h}_{i}$, ${\alpha v}_{i}$, and ${\alpha c}_{i}$ represent the differences between any two colors in a combination in terms of hue, value, and chroma, respectively. $A_{b_{i}}$ represents the value reflecting area balance, usually determined by the scalar moment of the colored region. When the number of colors in a combination is n, $A_{b_{i}}$ can be calculated using Equation (3).

(3) $$A_{b_{i}}=C\left(n,\;2\right)=n!/\left[2!\left(n-2\right)!\right]$$

Furthermore, when all colors in a combination are chromatic, Equation (2) can be used to determine the order (*O*) between two colors. However, if achromatic colors are present in the combination, the order between an achromatic color and any other color is always 1, i.e., *O* = 1.

Since Moon and Spencer introduced the quantitative model for color harmony (i.e., *M* = *O*/*C*), numerous scholars have utilized it for designing and evaluating product color matching, validating its effectiveness and scientific merit [7,37,38]. In this study, color harmony theory is employed in the color generation phase of the proposed design methodology to select the color combination with the highest aesthetic measurement, thereby laying the foundation for the product color-matching design phase.

#### 2.2.2. Color Matching Aesthetics Measurement

Based on the hue, value, and chroma values of colors within the Munsell color system, Hsiao et al. [16] introduced a quantitative model to determine the aesthetics of product color matching. This mathematical model has been applied by Lu and Hsiao [3] to assess the color aesthetics across different product types, demonstrating its effectiveness and practicality. In this quantitative model, in addition to considering the hue, value, and chroma of colors, the number of colors and their areas must also be taken into account. Once these parameters are obtained, the color-matching aesthetic measurement of a product ($M_{p}$) can be expressed as follows:

(4) $$M_{p}=\frac{\sum_{i=1}^{n}\left(U_{i}+Q_{i}\right)P_{i}}{n}$$

where *n* denotes the total count of colors used within a product; $U_{i}$ represents the contribution of color *i* (1 ≤ *i* ≤ n) to the aesthetics of product color matching, determined by its hue for chromatic colors or value for achromatic colors; $Q_{i}$ represents the contribution from the inverse product of chroma and value for color *i*, affecting the overall aesthetics of product color matching; $P_{i}$ represents the contribution of the area of color *i* to the aesthetics of product color matching. The detailed calculation methods for these parameters are described below.

**Figure 1:**
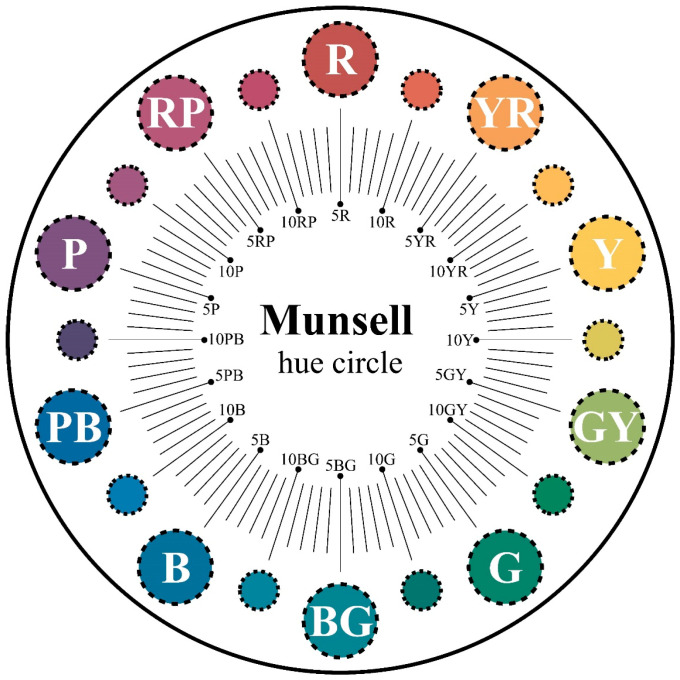
The Munsell hue circle.

**Figure 2:**
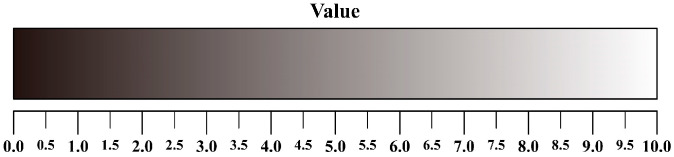
The value bar of colors.

For $U_{i}$, if color *i* is chromatic, the value of $U_{i}$ is determined by the hue of the color. Conversely, if color *i* is achromatic, the value of $U_{i}$ is determined by the value of the color. Therefore, the calculation formula for $U_{i}$ can be expressed as follows:

(5) $$U_{i}=\left\{\begin{array}{l} \begin{array}{cc} U_{h_{i}} & for\;a\;chromatic\;color \end{array}\\ \begin{array}{cc} U_{v_{i}} & for\;an\;achromatic\;color \end{array} \end{array}\right.$$

where $U_{h_{i}}$ represents the contribution of chromatic color *i* to the aesthetics of product color matching, and $U_{v_{i}}$ represents the contribution of achromatic color *i* to the aesthetics of product color matching. The calculation methods for both are described below. Before beginning the calculation, a sensibility evaluation based on target imagery should be conducted for the selected colors to determine their priority. Subsequently, if the color is chromatic, the specific calculation steps are as follows: first, divide the Munsell hue circle into 100 equal parts, using the 10 primary hues (Red (R), Yellow–Red (YR), Yellow (Y), Green–Yellow (GY), Green (G), Blue–Green (BG), Blue (B), Purple–Blue (PB), Purple (P), Red–Purple (RP)) as reference points (see Figure 1). Second, assign a hue value of 1 (i.e., $U_{h}$ = 1) to the color ranked first in the sensibility evaluation and a hue value of 0 (i.e., $U_{h}$ = 0) to the color ranked last. Then, divide the entire hue circle into two parts based on these two hue values (both starting from $U_{h}$ = 0 to $U_{h}$ = 1 in a clockwise and counterclockwise direction, respectively). Third, locate the hue value of the color used in product color matching on the hue circle, and use the proportion of the hue value within either part of the hue circle as the contribution of this chromatic color to the aesthetics of product color matching. If the color is achromatic, the specific calculation steps are as follows: first, divide the value bar into 100 equal parts (see Figure 2). Second, assign a value of 1 ($U_{v}$ = 1) to the color ranked first in the sensibility evaluation and a value of 0 ($U_{v}$ = 0) to the color ranked last. Then, divide the entire value bar into two parts (both starting from $U_{v}$ = 0 to $U_{v}$ = 1). Third, locate the value of the color used on the value bar, and use the proportion of the value within either part as the contribution of this achromatic color to the aesthetics of product color matching.

For $Q_{i}$, if color i is chromatic, its value is determined by the product of its chroma and value. Conversely, if color *i* is achromatic, its value is determined by its value. The specific calculation formula is given in Equation (6):

(6) $$Q_{i}=\left\{\begin{array}{l} \begin{array}{cc} \frac{\left(1/V_{i}C_{i}\right)}{\sum_{i=1}^{n}\left(1/V_{i}C_{i}\right)} & for\;a\;chromatic\;color \end{array}\\ \begin{array}{cc} \frac{\left(1/V_{i}\right)}{\sum_{i=1}^{n}\left(\left(1/V_{i}\right)\right)} & for\;an\;achromatic\;color \end{array} \end{array}\right.$$

where $V_{i}$ represents the value of color *i* and $C_{i}$ represents the chroma of color *i*.

For $P_{i}$, the number of pixels of each color is used as the area of that color. The value of $P_{i}$ can be calculated by dividing the area of color *i* by the area of the largest color, as shown in Equation (7):

(7) $$P_{i}=\frac{A_{i}}{max\underline{A}}$$

where $A_{i}$ represents the area of color *i* and $max\underline{A}$ represents the largest area among all the colors.

### 2.3. Macroscopic Aesthetic Evaluation of Shape Curves

Compared to the microscopic shape information of curves, such as circumference, maximum width, area, and roundness, Harada et al. [39] suggested from an industrial design perspective that humans can perceive not only the microscopic information of shape curves but also their macroscopic characteristics, such as curvature variation. To explore the connection between macroscopic shape information and the curve aesthetics, Ujiie et al. [40] introduced a method utilizing information theory, thermodynamic entropy, and the Markov process to assess the aesthetics of product shape curves, naming it quadratic curvature entropy (QCE).

Specifically, from the perspective of information theory, a product shape curve can be regarded as an information source, while its curvature can be seen as source symbols. The probability of these source symbols occurring can be defined as information content. The average information content represents the average uncertainty of the information, analogous to the concept of entropy in thermodynamics. Therefore, the curvature entropy of a shape curve can be expressed as follows:

(8) $$H=\sum_{\tau =1}^{\Lambda }p_{\tau }I_{\tau }=-\sum_{\tau =1}^{\Lambda }p_{\tau }{log}_{2}p_{\tau }$$

where *H* represents curvature entropy (i.e., average information content); *Iτ* denotes the information content; *pτ* represents the probability of source symbol *sτ* occurring; and Λ indicates the number of source symbols.

Building on curvature entropy, Ujiie et al. [40] further considered the interrelationships between source symbols to investigate the perception of macroscopic curvature variations. They proposed a Markov process-based curvature entropy, which is illustrated in Equation (9):

(9) $$H_{m}=\sum_{v=1}^{\Lambda^{\delta }}\sum_{\tau =1}^{\Lambda }q_{v}q_{v,\tau }I_{v,\tau }=-\sum_{v=1}^{\Lambda^{\delta }}\sum_{\tau =1}^{\Lambda }q_{v}q_{v,\tau }{log}_{2}q_{v,\tau }$$

where *q_v_* represents the probability of state occurrence; *δ* is the count of source symbols in the state; Λ represents the total number of symbol types; *q*_*v*,*τ*_ denotes the transition probability from source symbol *sv* to *sτ*; and *I*_*v*,*τ*_ represents the information content derived from the Markov process.

**Figure 3:**
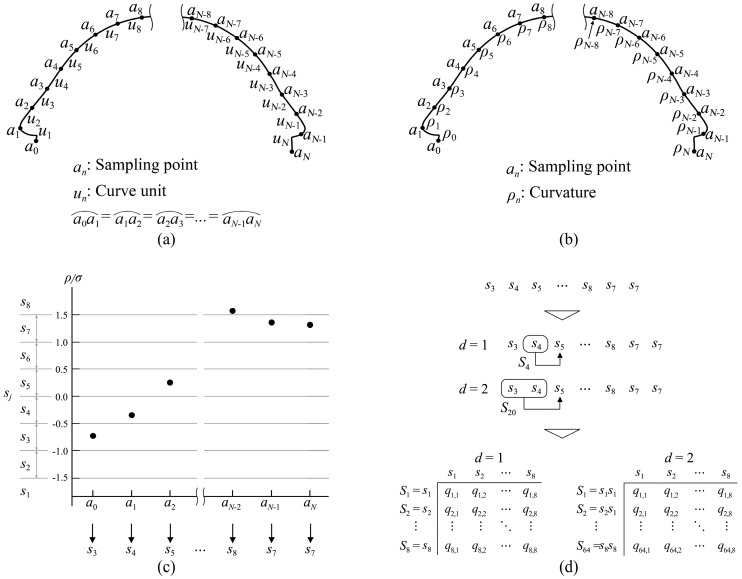
The calculation process of quadratic curvature entropy: (**a**) sampling point and curve unit; (**b**) sampling of the curvature; (**c**) quantization based on the curvature; (**d**) calculation of the transition probability.

As shown in Figure 3, considering the curvature of a shape curve as an information source, the steps to calculate curvature entropy using the Markov process are summarized as follows. First, in the Rhino environment, the shape curve is partitioned into *N* equal curve units based on sampling points (see Figure 3a), and the curvature *ρn* at each sampling point is obtained using a curve analysis tool (see Figure 3b). Second, the standard deviation of the curvature *σ* is calculated, and (*ρn*/*σ*) is used as the set of source symbols. Third, the range of (*ρn*/*σ*) values and the number of source symbol types *V* are determined, with the range set to [−1.5, 1.5] and *V* set to 8 (i.e., from s1 to s8) (see Figure 3c). Fourth, the occurrence probability of state *Si* (*q*_*i*_) and the transition probability from state *Si* to source symbol sj (*q*_*i*,*j*_) are calculated (see Figure 3d). Based on these steps, the curvature entropy calculated using the Markov process can be obtained. After normalization, it is referred to as QCE, as shown in Equation (10):

(10) $$H_{QC}=-\frac{1}{{log}_{2}V}\sum_{i=1}^{v^{d}}\sum_{j=1}^{V}q_{i}q_{i,j}{log}_{2}q_{i,j}\;(0\leq H_{QC}\leq 1)$$

where *d* represents the number of source symbols constituting a state. Moreover, to ensure that the QCE value falls between 0 and 1, the result is normalized by dividing it by the maximum entropy value log_2_*V*. Previous studies have confirmed that QCE can be used to evaluate the macroscopic aesthetics of product shape curves [5,41,42], indicating that the smaller the entropy value, the more aesthetically pleasing the shape curve is to human perception. In this paper, QCE is used to evaluate the shapes generated by image-GLMs to select the shape with the highest aesthetic value.

## 3. Implementation Procedures

### 3.1. Defining the Design Problem and Clarifying the Research Objectives

Tourism IP cultural products are goods developed based on intellectual property for specific tourist regions, attractions, or events, and have become an essential force in promoting tourism economic growth and cultural dissemination [43]. Hayden and Dills [44] suggested that tourism IP cultural products are diverse in form, which can be categorized as characters, animals, and symbolic objects. Su and Li [45] pointed out that tourism IP cultural products not only enrich visitors’ experiences but also enhance the development of the local tourism retail industry. Xu et al. [46] argued that high-quality tourism IP cultural products can create a tangible impression of attractions for tourists and contribute to tourism development. Specifically, tourism IP cultural products help convey local culture to visitors and encourage interaction [47]. Notable international examples include the “Bing Dwen Dwen” tourism IP cultural product from the Beijing 2022 Winter Olympics, which attracted significant attention from both domestic and international tourists [48], and the “Kumamon” tourism IP cultural product from Kumamoto City, which drew a large number of visitors [49].

Scholars have recently proposed various design methods to create appealing tourism IP cultural products. Liu et al. [11] proposed a design method for IP cultural products based on Kansei engineering and fuzzy theory. Kang and Nagasawa [50] developed a strategy for optimizing IP cultural product design using Kansei engineering and an interactive genetic algorithm. Liu et al. [51] emphasized that in developing tourism IP cultural products, it is essential not only to integrate local cultural characteristics but also to meet the diverse emotional needs of tourists. In these studies, imagery adjectives are often used to establish models that link tourists’ emotional data with IP cultural product design features, specifically color matching and shape. Additionally, as previously discussed, GLMs are increasingly becoming a new productivity tool in product design. Therefore, to promote tourism development, it is necessary to propose a design methodology for color matching and shape of tourism IP cultural products using GLMs. The research objectives for this design problem are as follows: (1) to propose a Kansei design methodology for tourism IP cultural products that integrates color matching and shape based on GLMs, thereby enhancing the efficiency of Kansei design for tourism IP cultural products; (2) to present a design case involving the collaboration of multimodal GLMs to inspire tourism IP cultural product designers to consider the efficient use of GLMs; and (3) to propose a quantitative evaluation method for GLM-generated content to improve the rigor and practicality of the GLMs-driven design methodology for tourism IP cultural products.

### 3.2. Planning the Implementation Procedures for the Design Methodology

**Figure 4:**
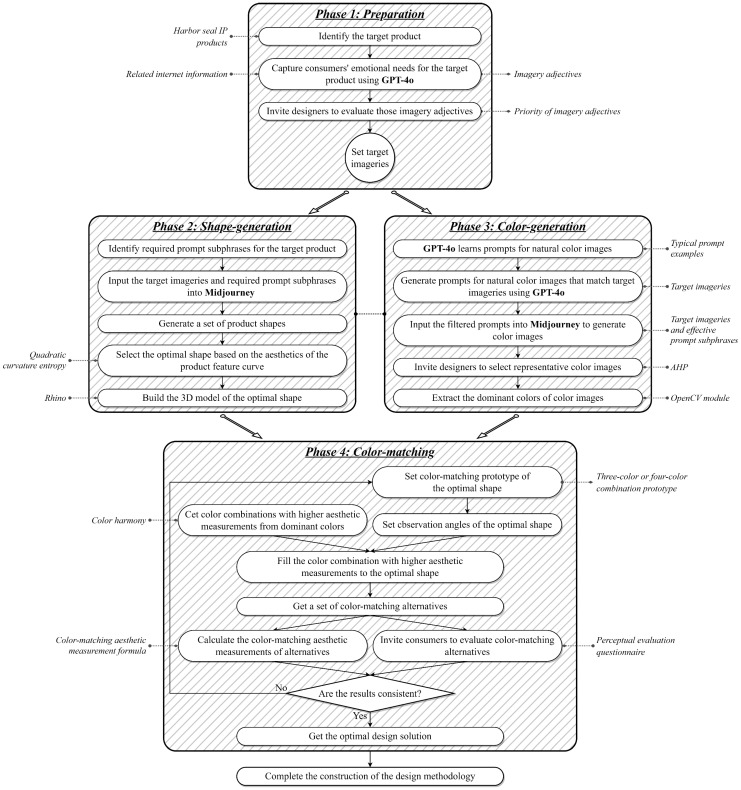
Flowchart of the generative large model-driven design methodology.

Dalian is a well-known coastal tourist city in China. The harbor seal, a unique marine species in Dalian, is also a Class I protected animal in China. Every year, a large number of tourists from both domestic and international locations visit Dalian to see the harbor seals. Therefore, this study takes the design of Harbor Seal IP cultural products as a case study to demonstrate the implementation procedures of the proposed design methodology and validate its effectiveness. Specifically, drawing on the theories and methodologies discussed in Section 2, this study integrates text-GLM, image-GLM, color harmony, color-matching aesthetic measurement, and quadratic curvature entropy to propose a design methodology consisting of the following four phases: preparation (Phase 1), shape generation (Phase 2), color generation (Phase 3), and color matching (Phase 4). Using the harbor seal as an example, the visual implementation process is illustrated in Figure 4, and the generalized implementation procedures are described as follows.

#### 3.2.1. Preparation (Phase 1)

Step 1: Define the target product and summarize consumers’ emotional needs into imagery adjectives using GPT-4o. First, select a symbolic or well-known object from a specific tourist region as the design prototype of an IP cultural product, and consider the evolved IP product as the target product. Then, collect online information about the target product and send its web links as prompts to GPT-4o. Finally, instruct GPT-4o to browse the web links and summarize the positive reviews of the target product into imagery adjectives. In practice, reviews of the target product can be obtained from online forums, special reports, and user comments.

Step 2: Evaluate the imagery adjectives and select the target imagery. Invite designers to participate in evaluating the imagery adjectives summarized by GPT-4o in Step 1 using a perceptual evaluation questionnaire. The evaluation criterion is whether each adjective is suitable as an imagery adjective to describe the target IP product. Based on reliability and validity analysis, prioritize the imagery adjectives according to the questionnaire results and set those with higher scores as the target imagery.

#### 3.2.2. Shape Generation (Phase 2)

Step 3: Generate numerous shapes that match the target imagery using Midjourney. First, use prompt engineering to define the required prompt subphrases for the target IP product. Typically, to use Midjourney to generate shapes that meet user expectations, essential prompt subphrases such as the target object, style type, and observation angle must be provided. Then, set the imagery adjectives as key prompt subphrases and position them as much as possible at the beginning of the prompt. Finally, merge the required prompt subphrases with the key prompt subphrases into a full prompt, then enter it into Midjourney using the “/imagine” command to generate shapes corresponding to the target imagery. The same prompt can be run multiple times to obtain a large number of effective shapes. In addition, to minimize the impact of other abstract terms on the generated shapes, other prompt subphrases should not contain any imagery tendencies.

Step 4: Select the optimal shape using QCE and construct its 3D model. First, invite designers to rate the shapes generated in Step 3 based on the target imagery, thereby selecting typical product shapes. Next, draw the main feature curves of the shape using cubic Bézier curves. Then, calculate the QCE for each feature curve based on the macroscopic aesthetic evaluation method described in Section 2.3. Finally, the shape with the lowest QCE is selected as optimal, and its 3D model is constructed using the Rhino CAD tool. Moreover, when creating the 3D model, the shape’s ergonomics and dimensions should be further optimized and adjusted, considering the product type.

#### 3.2.3. Color Generation (Phase 3)

Step 5: Generate prompts for natural color images using GPT-4o based on prompt examples and select effective prompt subphrases. First, collect quality prompt examples for natural color images from the Discord platform and the Promptalot website. Then, instruct GPT-4o to learn from the collected prompt examples and generate several new prompts that match the target imagery. Finally, select effective prompt subphrases from the generated prompts. Additionally, Midjourney’s “/describe” command can convert natural color images into prompt examples, serving as learning examples for GPT-4o.

Step 6: Generate numerous natural color images using Midjourney and select representative images based on the analytic hierarchy process (AHP). Input the effective prompt subphrases and key prompt subphrases (i.e., target imageries) into Midjourney’s “/imagine” command. Running the prompt multiple times will generate numerous natural color images. Subsequently, invite designers to use AHP to select representative color images from the generated images based on the target imagery.

Step 7: Use the OpenCV module to extract the dominant colors from the representative color images. Write an interactive program with the OpenCV module in Python to extract colors from the images. Input the color images into the program to obtain the dominant colors. Specifically, the program calculates the area occupied by each color and identifies the colors with the largest areas as the dominant colors.

#### 3.2.4. Color Matching (Phase 4)

Step 8: Define the color-matching prototype and observation angles for the optimal shape. Divide the coloring areas based on factors such as the target product’s functional characteristics, shape structure, material, and surface treatment process, thereby forming a color-matching prototype. The color-matching prototype specifies the number of colors used in the color-matching scheme. In addition, suitable observation angles must be set for the target product to facilitate subsequent aesthetic measurements of color matching. The observation angles should reveal as much of the shape information as possible. In practice, different color-matching prototypes and multiple observation angles can be set for the target product.

Step 9: Calculate aesthetically pleasing color combinations from the dominant colors based on color harmony. The color-matching prototype set in Step 8 determines the number of colors in the combination (e.g., three-color or four-color combination). Then, use a pseudorandom approach to reorganize the dominant colors obtained in Step 7 into a series of color combinations. Finally, calculate the aesthetic measurements for all color combinations using Equations (1) to (3) described in Section 2.2.1, and select the combinations with higher aesthetic values.

Step 10: Generate a collection of color-matching alternatives. Fill the color combinations with higher aesthetic values obtained in Step 9 into the color-matching prototype set in Step 8 to obtain a collection of color-matching alternatives. When filling the colors, some basic visual aesthetic principles should be followed, such as placing colors with lower value at the bottom coloring area to create visual stability.

Step 11: Evaluate the alternatives using the color-matching aesthetic quantification formula and a perceptual evaluation questionnaire. Following the calculation steps outlined in Section 2.2.2, apply Equations (4) to (7) to determine the aesthetic measurements of color matching for the alternatives. Additionally, consumers will be invited to evaluate alternatives based on target imagery through a perceptual evaluation survey. In practice, an interactive calculation program can be developed to improve the efficiency of calculating the aesthetic measurements of color matching.

Step 12: Conduct a correlation analysis of the evaluation results and select the optimal design solution. Use Pearson correlation analysis to assess the relationship between the two sets of evaluation results obtained in Step 11. If the correlation is significant, the alternative with the highest score is chosen as the optimal design solution. Otherwise, analyze other potential factors influencing the evaluation results and return to the initial step of this phase.

## 4. Case Study

Following the implementation steps planned in Section 3.2 and the visual implementation process (Figure 4), this section uses the Harbor Seal IP cultural product design as a case study to address the design problem defined in Section 3.1 and achieve the proposed research objectives.

### 4.1. Phase 1: Preparation

#### 4.1.1. Capturing Consumers’ Emotional Needs for Harbor Seal IP Products

News’ reports and comments related to the harbor seal were collected from online sources (i.e., special reports and social media) and their links were sent to GPT-4o. GPT-4o was then instructed to analyze the positive content of these online comments and summarize them into abstract adjectives. These adjectives represent tourists’ emotional perceptions of the harbor seal and can be used as references for designing the harbor seal IP product. Specifically, these adjectives serve as the basis for Kansei design of the product’s shape and color matching. GPT-4o ultimately summarized 14 imagery adjectives, namely, dreamy, healing, cute, noble, unique, delicate, dazzling, attractive, cyberpunk, girly, lively, artistic, interesting, and technological.

#### 4.1.2. Setting Target Imageries for Harbor Seal IP Products

Shape and color matching are essential aspects of the design features for IP products. To select suitable adjectives from the 14 imagery adjectives describing the shape and color matching of harbor seal IP products, 20 product designers were asked to assess these adjectives. The evaluation criterion was whether each adjective was appropriate for describing the shape and color matching of the harbor seal IP product, rated on a scale from 0 to 1. The average score from all designers was taken as the effective score, and the top seven adjectives are shown in Table 3. Statistical analysis showed a Cronbach’s α of 0.85, indicating reliable questionnaire results. In this study, the top two adjectives were selected as the target imageries for the Harbor Seal IP product, namely healing and cyberpunk.

### 4.2. Phase 2: Shape Generation

#### 4.2.1. Setting Prompt for Harbor Seal IP Products

The Harbor Seal IP product prompt consisted of required prompt subphrases and key prompt subphrases. The required prompt subphrases included the harbor seal IP product, style type, and observation angle, while the key prompt subphrases were the target imageries of healing and cyberpunk. As Midjourney prioritizes prompt subphrases that appeared earlier, the key prompt subphrases were placed in the second position. Additionally, the aspect ratio of the output image was set to 3:4, and the model type used was “niji”. The complete prompt was the following: {Harbor Seal IP product, healing/cyberpunk, blind box, POP MART, C4D, front view, high quality, 8K, white background, no color --ar 3:4 --niji 5 --style expressive}.

#### 4.2.2. Generating Harbor Seal IP Shapes Corresponding to Target Imageries

**Figure 5:**
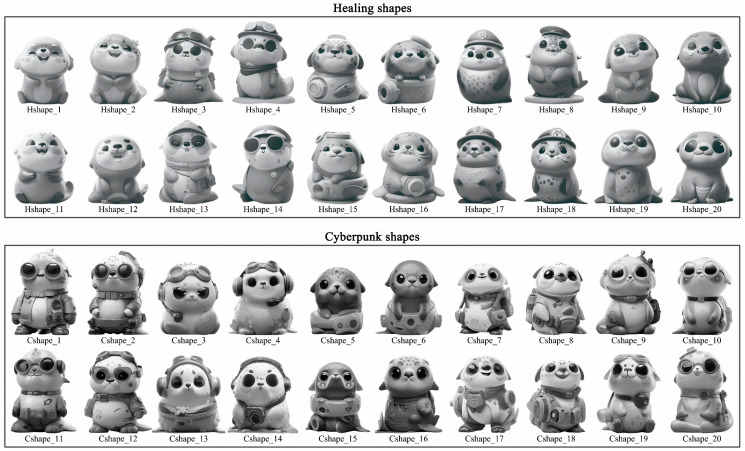
An interface for generating Harbor Seal IP shapes using Midjourney.

The prompt was entered into Midjourney’s “/imagine” command to generate harbor seal IP product shapes reflecting the target imageries (i.e., healing and cyberpunk). After multiple runs, 20 shapes were created for each target imagery, as illustrated in Figure 5. Hshape_1 to Hshape_20 represent the healing shapes, while Cshape_1 to Cshape_20 represent the cyberpunk shapes. Subsequently, to select shapes that accurately correspond to the target imageries, 20 product designers were invited to evaluate the generated shapes based on the target imagery and select 10 typical shapes. Statistical analysis showed that the top 10 healing shapes were Hshape_1, Hshape_2, Hshape_6, Hshape_7, Hshape_9, Hshape_10, Hshape_11, Hshape_16, Hshape_19, and Hshape_20, while the top 10 cyberpunk shapes were Cshape_1, Cshape_2, Cshape_4, Cshape_5, Cshape_6, Cshape_8, Cshape_14, Cshape_17, Cshape_18, and Cshape_20.

#### 4.2.3. Selecting the Optimal Shape Based on Macroscopic Aesthetic Evaluation of Shape Curves

**Figure 6:**
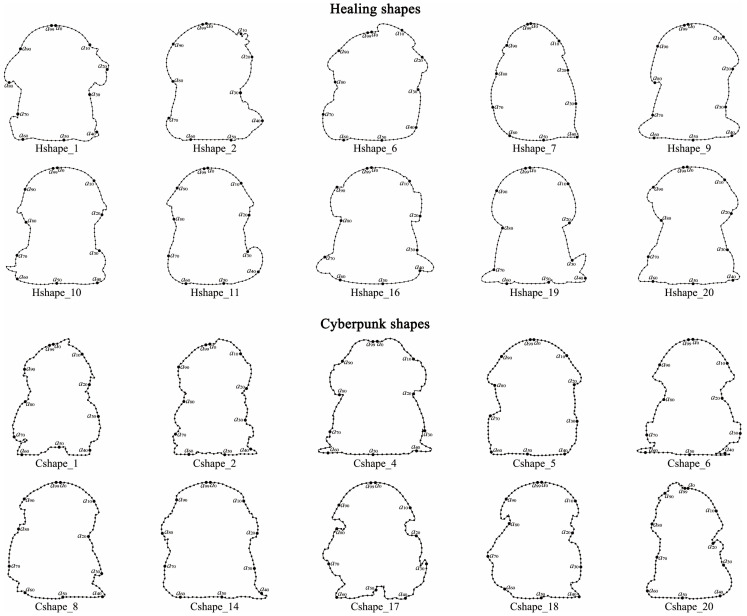
Feature curves of healing and cyberpunk shapes.

**Figure 7:**
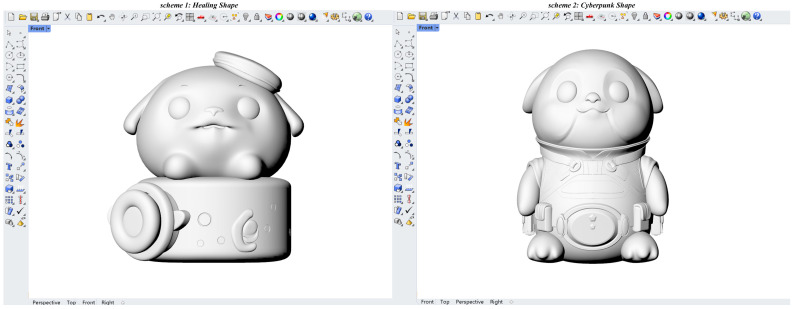
The three-dimensional models of the two optimal shape schemes.

In the previous section, 10 typical shapes were selected from 20 shapes for each target imagery. This section aims to select the shape with the highest aesthetic value as the optimal shape based on the macroscopic aesthetics of shape curves. First, the feature curves of the 10 typical shapes were drawn using differentiable cubic Bézier curves. Second, each feature curve was divided into 100 equal curve units using 100 sampling points (see Figure 6). Finally, the entropy value of each feature curve was calculated based on the QCE calculation steps described in Section 2.3 and Equation (10), as shown in Table 4. The results indicated that the healing shape Hshape_6 had the lowest entropy value (*H_QC_* = 0.241), while the cyberpunk shape Cshape_8 had the lowest entropy value (*H_QC_* = 0.278). Therefore, Hshape_6 and Cshape_8 were considered the optimal shapes. Additionally, since the entropy value of Hshape_19 was only 0.001 higher than that of Hshape_6, it can be considered a reserve option for the optimal healing shape in practical applications. Subsequently, 3D models were constructed for Hshape_6 and Cshape_8 using Rhino, with optimization of ergonomic dimensions. The 3D models of the two optimal shapes are shown in Figure 7.

### 4.3. Phase 3: Color Generation

#### 4.3.1. Generating Natural Color Images Corresponding to Target Imageries

**Figure 8:**
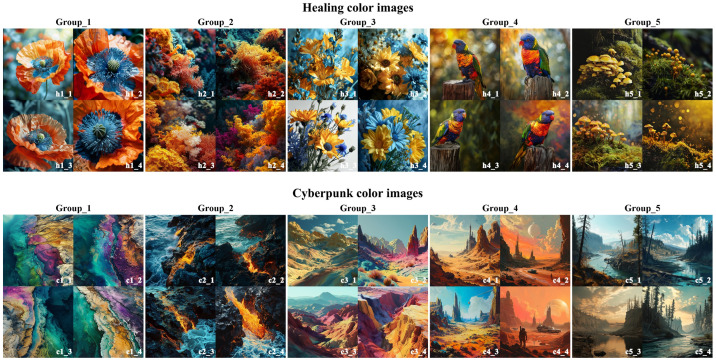
Healing and cyberpunk color images generated by Midjourney.

In this section, natural color images corresponding to the target imageries were generated through collaboration between a text-GLM (i.e., GPT-4o) and an image-GLM (i.e., Midjourney). First, quality prompt examples for natural color images were collected from various open prompt-generation platforms. Additionally, high-quality natural color images were converted into prompt examples using the “/describe” command in Midjourney. Second, the collected prompt examples were provided to GPT-4o to learn the structure and phrasing of these prompts. A total of 24 prompt examples were gathered. Third, GPT-4o was instructed to generate five new prompts for natural color images corresponding to the target imageries based on the 24 prompt examples. Effective prompt subphrases were then selected from the generated prompts and, together with the target imageries, were input into Midjourney’s “/imagine” command to generate five sets of natural color images (see Figure 8).

#### 4.3.2. Selecting Representative Natural Color Images Based on AHP

This section selected representative natural color images from Figure 8 using the AHP. First, 12 designers were invited to construct pairwise comparison matrices between the five sets of natural color images for each target imagery, using the target imagery as the evaluation criterion, as shown in Table 5 and Table 6. Among the five sets for the healing imagery, group_3 had the highest weight, making it the optimal group. Among the five sets for the cyberpunk imagery, group_4 had the highest weight, making it the optimal group. Second, designers were again invited to construct pairwise comparison matrices for the four color images in group_3 of the healing color images and group_4 of the cyberpunk color images, using the target imagery as the evaluation criterion, as shown in Table 7 and Table 8. In group_3 of the healing color images, h3_1 had the highest weight, making it the representative color image. In group_4 of the cyberpunk color images, c4_3 had the highest weight, making it the representative color image. Consistency checks showed that all consistency ratio (*C.R.*) values for the pairwise comparison matrices were less than 0.1, indicating acceptable evaluation results.

#### 4.3.3. Extracting Dominant Colors from Representative Color Images

**Figure 9:**
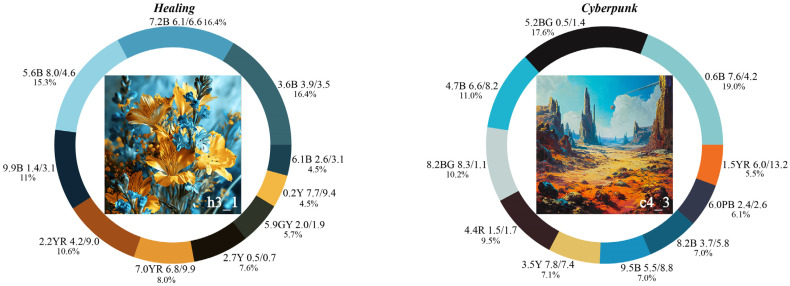
Dominant colors in representative color images of healing and cyberpunk.

To identify the dominant colors in the representative color images, an interactive program was created using the OpenCV module in Python to calculate the area occupied by each color in the images. Specifically, h3_1 and c4_3 were input into the program one by one to obtain the hue, value, and chroma (HVC) values of the top ten colors by area, which were defined as the dominant colors of the representative color images. The final results are presented in Figure 9.

### 4.4. Phase 4: Color Matching

#### 4.4.1. Setting Color-Matching Prototype and Observation Angles for the Optimal Shape

**Figure 10:**
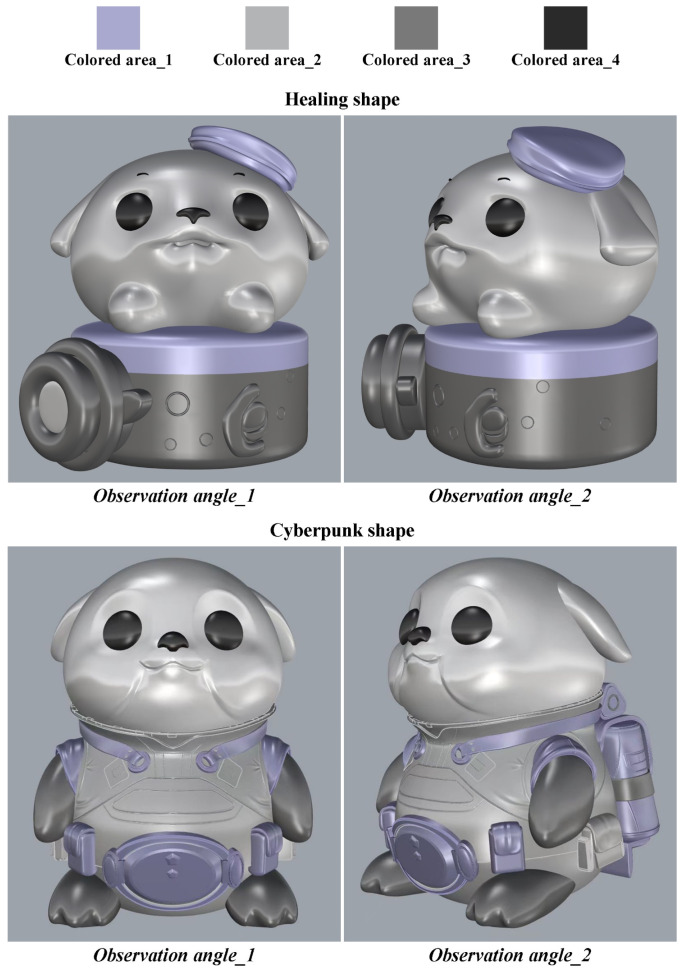
Color-matching prototype and observation angles for the Harbor Seal IP product.

This section sets the color-matching prototype and observation angles for the optimal shapes obtained in Section 4.2.3 (see Figure 7). In this study, the prototype of the target product is an animal (i.e., harbor seal), whose characteristics have been anthropomorphized into an IP cultural product. Therefore, when setting the color-matching prototype, both functional, structural, material, and surface treatment aspects of the product shape and the inherent biological features of the harbor seal were considered. Based on these influencing factors, the color-matching prototypes of the optimal shapes for healing and cyberpunk are shown in Figure 10. The color-matching prototypes include four colored areas (i.e., colored area_1, colored area_2, colored area_3, and colored area_4), indicating that the subsequent color matching requires four-color combinations. Subsequently, two observation angles were set to comprehensively present the harbor seal IP product (i.e., observation angle_1 and observation angle_2). Observation angle_1 is the front view of the harbor seal IP product, containing the complete shape information, while observation angle_2 is a perspective view that supplements the missing shape information from the front view. Compared to color-matching evaluation based on a single observation angle [7], using two observation angles is more rigorous.

#### 4.4.2. Obtaining Four-Color Combinations with Higher Aesthetic Measurements from the Dominant Colors

**Figure 11:**
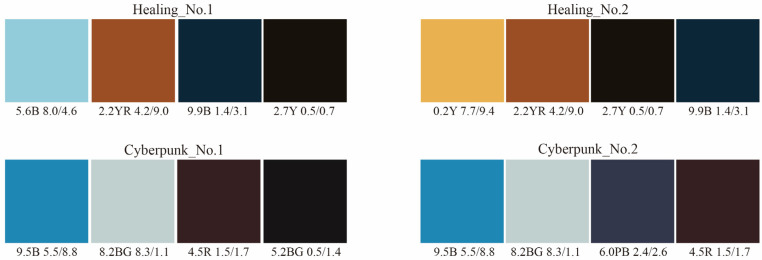
The top two four-color combinations with the highest aesthetic measurement.

Theoretically, the ten dominant colors in Figure 9 can form 210 four-color combinations, resulting in 420 combinations from the two sets of dominant colors. Based on the calculation steps described in Section 2.2.1 and Equations (1) to (3), the color harmony aesthetic measurement of each four-color combination was calculated. This study selected the top two four-color combinations with the highest aesthetic measurements for color matching. The top two four-color combinations are shown in Figure 11, and the relevant calculation parameters are presented in Table 9.

#### 4.4.3. Obtaining Color-Matching Alternatives

**Figure 12:**
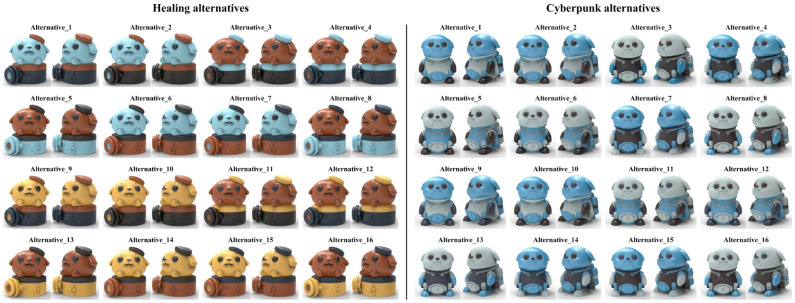
Color-matching alternatives for healing and cyberpunk.

The four-color combinations combination_1 and combination_2 were applied to the two color-matching prototypes of the optimal healing shape, while combination_3 and combination_4 were applied to the two color-matching prototypes of the optimal cyberpunk shape. Considering the anthropomorphized shape features of the harbor seal IP product, the achromatic color or the chromatic color with the lowest value from the four-color combination were filled into the eye area. Given this constraint, one four-color combination could generate eight color-matching alternatives. Combination_1 and combination_2 generated 16 healing alternatives, while combination_3 and combination_4 generated 16 cyberpunk alternatives. Subsequently, the KeyShot was used to render the alternatives with realistic lighting effects, as shown in Figure 12.

#### 4.4.4. Evaluating Color-Matching Alternatives

The alternatives shown in Figure 12 were evaluated using both the color-matching aesthetic quantification formula and a perceptual evaluation questionnaire.

**Figure 13:**
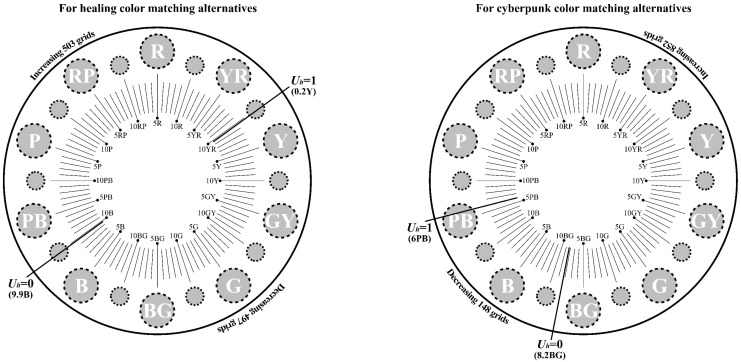
Schematic diagram of calculating the contribution of hue in chromatic colors to color-matching aesthetic measurement.

**Figure 14:**
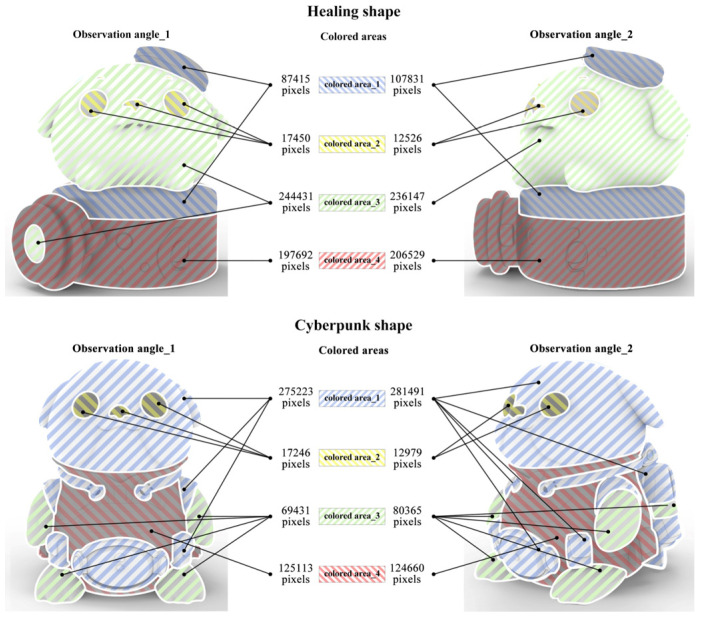
The area of each colored area in the shapes of healing and cyberpunk from two observation angles.

**Figure 15:**
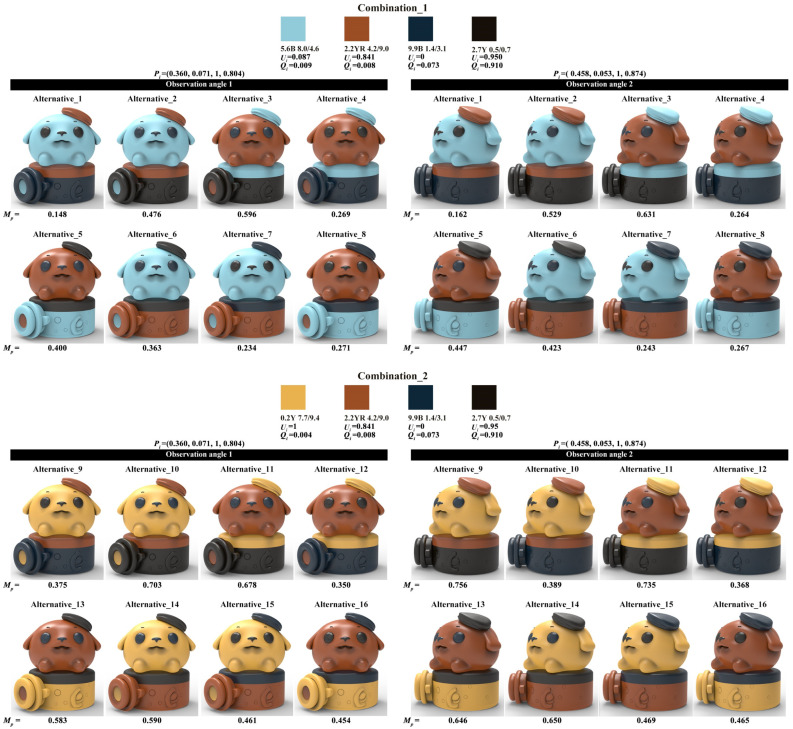
Calculation results of color-matching aesthetic measurement of healing alternatives.

**Figure 16:**
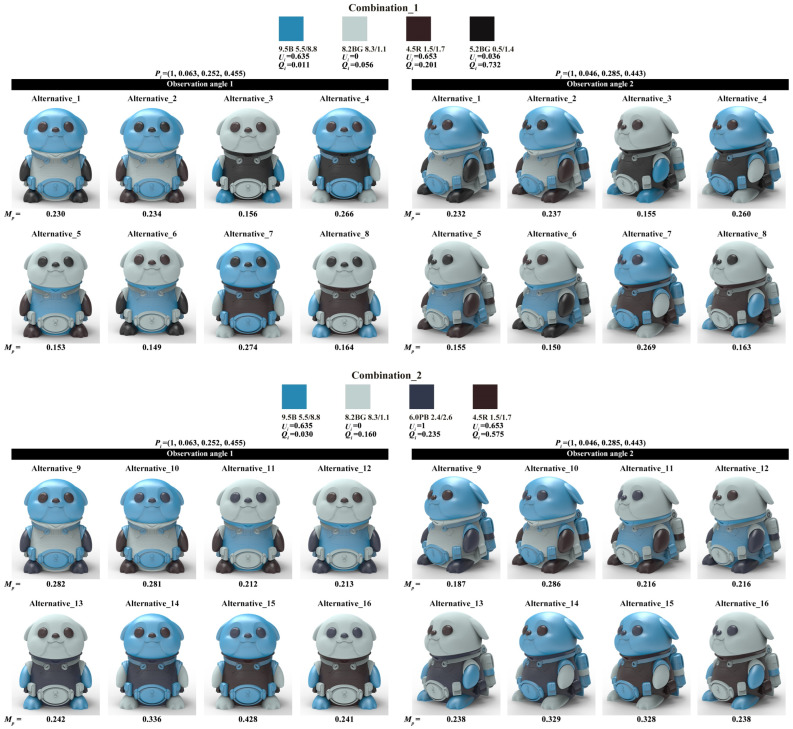
Calculation results of color-matching aesthetic measurement of cyberpunk alternatives.

For quantitative evaluation, Equations (4) to (7) introduced in Section 2.2.2 were used to calculate $U_{i}$, $Q_{i}$, and $P_{i}$ successively, ultimately obtaining the color-matching aesthetic measurement (*M_p_*) for each alternative. First, students and teachers specializing in design were invited to rate the five healing colors and five cyberpunk colors in Figure 11 based on the criteria of “How healing is this color?” and “How cyberpunk is this color?” The evaluation results showed that the first and last of the five healing colors were 0.2Y 7.7/9.4 and 9.9B 1.4/3.1, respectively; for the cyberpunk colors, they were 8.2BG 8.3/1.1 and 6.0PB 2.4/2.6. The hue values of the first and last colors were used to divide the hue circle into two parts (see Figure 13), and the contribution of each chromatic color to the color-matching aesthetic measurement ($U_{i}$) was determined accordingly. Second, the value and chroma of the colors were substituted into Equation (6) to determine their contribution to the color-matching aesthetic measurement ($Q_{i}$). Third, Photoshop was used to display the number of pixels in each colored area, which was taken as the area of that colored area (see Figure 14). The area parameter was then substituted into Equation (7) to determine the contribution of the area to the color-matching aesthetic measurement ($P_{i}$). Finally, $U_{i}$, $Q_{i}$, and $P_{i}$ were substituted into Equation (4) to obtain the aesthetic measurement (*M_p_*) for each color-matching alternative. The calculation results are shown in Figure 15 and Figure 16, and the statistical results are presented in Table 10 and Table 11. After summing the aesthetic measurements for the two observation angles, Alternative_11 had the highest score among all healing alternatives (*M_p_* = 1.413), and Alternative_15 had the highest score among all cyberpunk alternatives (*M_p_* = 0.756). Therefore, based on the quantitative evaluation results, Alternative_11 and Alternative_15 were considered the optimal design solutions.

For qualitative evaluation, tourists were invited to assess the color-matching alternatives shown in Figure 12. The evaluation criterion was “How healing/cyberpunk is the color matching of this harbor seal IP cultural product?” on a scale from 0 to 1. Out of 140 distributed questionnaires, 128 valid responses were received. Statistical analysis yielded a Cronbach’s α value of 0.82, demonstrating acceptable questionnaire results. Table 12 and Table 13 present the average scores for each alternative under the two observation angles and their total scores. Regarding the total score, Alternative_14 received the highest score (score = 1.64), and Alternative_15 achieved the highest score (score = 1.69). Consequently, based on the qualitative evaluation results, Alternative_14 and Alternative_15 were considered the optimal design solutions.

Furthermore, Pearson correlation analysis was used to verify whether there was a correlation between the quantitative and qualitative evaluation results. Specifically, the total aesthetic measurements from Table 10 and Table 11 and the total scores from Table 12 and Table 13 were used as analysis data. Statistical analysis showed that the correlation coefficients for both the healing and cyberpunk alternatives were greater than 0.7, with *p*-values less than 0.05, indicating a significant correlation between the quantitative and qualitative evaluation results (see Table 14). Overall, both Alternative_11 and Alternative_14 ranked among the top in both evaluations with little difference, making either of them suitable as the optimal healing design solution. Alternative_15 ranked first in both evaluations, making it unquestionably the optimal cyberpunk design solution.

## 5. Results and Discussion

Section 4 implemented and validated the design methodology based on GLMs using the Harbor Seal IP cultural product example. The first three phases of the methodology (i.e., preparation, shape generation, and color generation) utilized the text-GLM GPT-4o and the image-GLM Midjourney. In the preparation phase, GPT-4o analyzed tourists’ emotional needs for the Harbor Seal IP cultural product and summarized them into 14 imagery adjectives through prompt engineering. Compared to traditional methods for collecting Kansei imageries, the intelligent search method based on GPT-4o can quickly gather tourists’ emotional feedback from the provided online information and summarize it into imagery adjectives, thereby reducing user research costs. In the shape generation phase, Midjourney was used to generate shapes for the Harbor Seal IP cultural product that corresponded to the target imagery, thereby forming a shape database (see Figure 5). Compared to traditional Kansei design methods for product shape, the shape generation method based on Midjourney can transform tourists’ abstract Kansei needs into a concrete product shape, laying the foundation for detailed shape design. In the color generation phase, GPT-4o served as the prompt generator for Midjourney, while Midjourney was used to create colored images aligned with the target imagery (see Figure 8). Compared to traditional methods of extracting colors from images for color matching, the color image generation method based on multimodal GLMs can transform abstract target imageries into corresponding natural color images, laying the foundation for color matching design. Overall, in this paper, a Kansei design methodology is presented for both the shape and color matching of tourism IP cultural products based on GLMs, using the Harbor Seal IP cultural product as an example to demonstrate a collaborative design case involving multimodal GLMs. It highlights the empowerment of generative AI for tourism IP cultural product design, promoting the intelligent development of such products.

**Figure 17:**
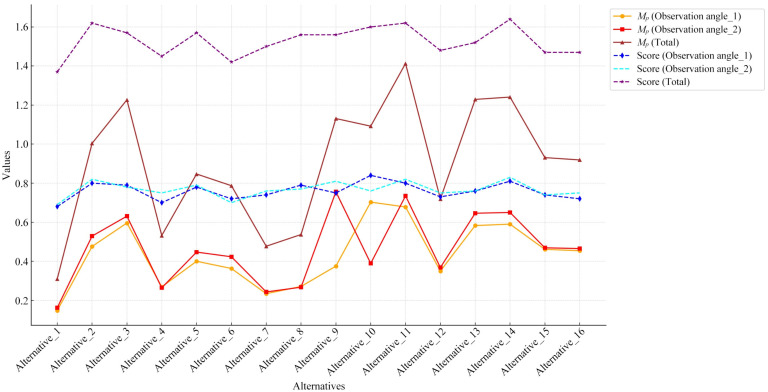
Line chart of scores for 16 healing alternatives based on quantitative and qualitative evaluation methods.

**Figure 18:**
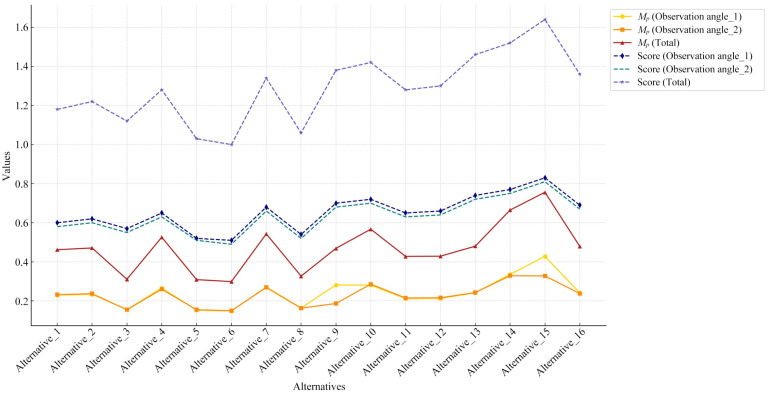
Line chart of scores for 16 cyberpunk alternatives based on quantitative and qualitative evaluation methods.

This paper emphasizes the quantitative evaluation process for content generated by GLMs. To select the optimal shape and representative color images from the GLM-generated content, this study employed quantitative evaluation methods based on QCE and AHP. Specifically, QCE was used as an indicator for evaluating shape aesthetics, where the lower the entropy value of the shape curve, the higher the aesthetic measurement. AHP was used to evaluate whether the color images corresponded to the target imageries, where higher weight values indicated better alignment with the target imagery. The quantitative evaluation not only improved the practicality of the design methodology but also ensured its rigor and scientific basis. In addition, a combination of quantitative and qualitative evaluations was used to select the optimal color-matching solution. The quantitative evaluation applied the color-matching aesthetic measurement formula to determine the aesthetic measurement of alternatives from two observation angles (see Table 10 and Table 11). In the qualitative evaluation, a perceptual evaluation questionnaire for tourists was used to obtain scores for the alternatives from two observation angles (see Table 12 and Table 13). Figure 17 presents the scores of the 16 healing alternatives in both quantitative evaluation (i.e., Mp (Observation angle_1), Mp (Observation angle_2), and Mp (Total)) and qualitative evaluation (i.e., score (Observation angle_1), score (Observation angle_2), and score (total)), while Figure 18 shows the scores for the 16 cyberpunk alternatives. In this paper, Pearson correlation analysis was conducted between the total values of the quantitative evaluation (i.e., Mp (Total)) and the qualitative evaluation (i.e., score (total)) (see Table 14). The results showed that the correlation coefficients were greater than 0.7, indicating a statistically significant correlation between the quantitative and qualitative evaluation results. Therefore, the optimal design solutions were selected based on a comprehensive consideration of both evaluation results.

## 6. Conclusions

This paper focuses on tourism IP cultural products and proposes an IP product color-matching and shape design methodology building on GLMs, using tourists’ emotional needs as a medium. The Harbor Seal IP product served as a case study, following the process of “preparation—shape generation—color generation—color matching” to demonstrate the implementation of the proposed methodology and validate its effectiveness through both quantitative and qualitative means. Compared with the literature in Table 1, this study makes the following academic contributions: (1) unlike existing design methodologies, the proposed GLM-based methodology considers both color matching and shape, promoting the intelligent development of Kansei design; (2) the different design phases of the proposed methodology utilized text and image-generative large models, presenting a collaborative design case involving multimodal GLMs and inspiring designers to explore the potential applications of other GLMs; and (3) a quantitative evaluation method for GLM-generated content based on the macroscopic shape information of shape curves was presented, enhancing the scientific rigor and practicality of GLM-driven design methodologies. In summary, generative large models improve the efficiency of Kansei design for product color matching and shape, paving a new path for intelligent product design.

Although this paper used the Harbor Seal IP cultural product as a case study to demonstrate the implementation of the proposed methodology and validate its effectiveness through quantitative and qualitative evaluations, some research limitations remain. First, the optimal shape was selected from those generated by the image-generative large model based on QCE and expert perceptual questionnaires; however, some scholars may argue that this approach overly relies on the generative model, thus reducing the designer’s subjective involvement. In future research, a shape elements chart could be constructed based on the generated shapes, and shape elements could be recombined into new shapes under other constraints. Second, this paper introduced a QCE-based method for evaluating shape aesthetics, but the evaluation only considered the external contour curves of the product shape without including other internal shape elements. Although the external contour is the most representative shape curve of tourism IP cultural products, future research should selectively consider representative shape curves based on the morphological characteristics of the target product to ensure scientific rigor in shape evaluation. Third, this study used the top ten colors by area ratio in color images as dominant colors to represent their overall imagery. However, colors with higher chroma and value also significantly contribute to the overall imagery of color images. Therefore, future research should develop a more rigorous strategy for selecting dominant colors to improve the feasibility of color imagery transfer. Finally, compared to other functional products, IP cultural products are more akin to decorative items. While their development does not require extensive consideration of functional components, technical design aspects such as material selection and manufacturing processes remain crucial. Therefore, future research could incorporate a technical design phase into the current framework to enhance the feasibility and manufacturability of product designs.

## Figures and Tables

**Table 1:** Several studies on the Kansei design of product shape and color matching.

Year	Author	Contribution	Object	Case
2015	Lo et al.	A product Kansei shape design and evaluation method was introduced, integrating a genetic algorithm with fuzzy theory [10].	shape	pushpin and stereo
2019	Liu et al.	A product Kansei shape design method was proposed, leveraging factor analysis and triangular fuzzy numbers [11].	shape	cultural creative product
2021	Zhou et al.	A product Kansei shape design and evaluation method was developed using convolutional neural networks [12].	shape	car
2023	Wu et al.	A product Kansei shape design and evaluation framework was established based on generative adversarial networks [13].	shape	car
2024	Yuan et al.	A product Kansei shape design method was proposed, combining eye-tracking technology with a genetic algorithm [6].	shape	electric vehicle
2024	Lu et al.	A product Kansei shape design method was proposed based on the form aesthetics theory and finite structure method [14].	shape	leafless fan
2007	Tsai and Chou	A product Kansei color-matching design method was introduced, employing a genetic algorithm and color harmony theory [15].	color	thermos flask
2008	Hsiao et al.	A method for quantifying the product Kansei color-matching aesthetics was presented [16].	color	cell phone
2013	Hsiao et al.	The effectiveness of the product Kansei color-matching quantification method was subsequently verified [17].	color	motorcycle
2015	Hsiao and Tsai	A method for using colors in color images for product Kansei color-matching design was proposed [18].	color	shoe
2022	Lu and Hsiao	A product Kansei shape and color-matching design method was developed by considering the product’s observation angles [3].	shape and color	vehicle and leafless fan
2024	Wu et al.	A generative artificial intelligence-enabled product Kansei design method was introduced [7].	shape and color	vacuum cleaner

**Table 2:** Quantitative intervals and their corresponding scores for the six types of relationships between any two colors in a color combination in hue, value, and chroma.

Comfortable Interval	Uncomfortable Interval	Hue		Value		Chroma	
		Difference	Score	Difference	Score	Difference	Score
Identity		0	1.5	0	−1.3	0	0.8
	First ambiguity	0–7	0	0–0.5	−1	0–3	0
Similarity		7–12	1.1	0.5–1.5	0.7	3–5	0.1
	Second ambiguity	12–28	0.65	1.5–2.5	−0.2	5–7	0
Contrast		28–50	1.7	2.5–10	3.7	>7	0.4
	Glare	–	–	>10	−0.2	–	–

**Table 3:** The top seven imagery adjectives.

Imagery Adjectives	Average Score
Healing	0.86
Cyberpunk	0.84
Cute	0.82
Delicate	0.77
Lively	0.74
Dazzling	0.71
Dreamy	0.71

**Table 4:** QCE values for healing and cyberpunk shapes.

Healing Shape Numbers	QCE Values	Cyberpunk Shape Numbers	QCE Values
Hshape_1	0.256	Cshape_1	0.319
Hshape_2	0.312	Cshape_2	0.338
Hshape_6	0.241	Cshape_4	0.363
Hshape_7	0.301	Cshape_5	0.288
Hshape_9	0.278	Cshape_6	0.321
Hshape_10	0.254	Cshape_8	0.278
Hshape_11	0.289	Cshape_14	0.368
Hshape_16	0.318	Cshape_17	0.348
Hshape_19	0.242	Cshape_18	0.327
Hshape_20	0.359	Cshape_20	0.357

**Table 5:** Pairwise comparison matrix of the five groups of healing color images.

	Group_1	Group_2	Group_3	Group_4	Group_5	Geometric Mean	Weight	Ranking
Group_1	1	0.864	0.792	1.056	1.218	0.974	0.19	3
Group_2	1.158	1	0.917	1.222	2.294	1.244	0.24	2
Group_3	1.263	1.091	1	1.333	3.112	1.417	0.27	1
Group_4	0.948	0.818	0.750	1	1.059	0.908	0.18	4
Group_5	0.821	0.436	0.321	0.941	1	0.641	0.12	5
$\lambda_{max}=5.069,\;C.R.=0.015<0.1⟹Accept$								

**Table 6:** Pairwise comparison matrix of the five groups of cyberpunk color images.

	Group_1	Group_2	Group_3	Group_4	Group_5	Geometric Mean	Weight	Ranking
Group_1	1	0.762	1.231	0.503	0.696	0.800	0.15	4
Group_2	1.313	1	1.615	0.778	0.913	1.085	0.21	3
Group_3	0.813	0.619	1	0.374	0.441	0.608	0.11	5
Group_4	1.988	1.286	2.677	1	1.174	1.517	0.29	1
Group_5	1.438	1.095	2.269		1	1.249	0.24	2
$\lambda_{max}=5.010,\;C.R.=0.002<0.1⟹Accept$								

**Table 7:** Pairwise comparison matrix of the four color images in the group_3 of healing color images.

	h3_1	h3_2	h3_3	h3_4	Geometric Mean	Weight	Ranking
h3_1	1	1.143	2.786	1.831	1.554	0.36	1
h3_2	0.875	1	2.145	1.077	1.192	0.28	2
h3_3	0.359	0.466	1	0.642	0.573	0.14	4
h3_4	0.546	0.929	1.557	1	0.943	0.22	3
$\lambda_{max}=4.015,\;C.R.=0.010<0.1⟹Accept$							

**Table 8:** Pairwise comparison matrix of the four color images in the group_4 of cyberpunk color images.

	c4_1	c4_2	c4_3	c4_4	Geometric Mean	Weight	Ranking
c4_1	1	1.095	0.622	1.211	0.953	0.22	2
c4_2	0.913	1	0.423	1.105	0.808	0.19	3
c4_3	1.609	2.362	1	2.947	1.829	0.42	1
c4_4	0.826	0.905	0.339	1	0.710	0.17	4
$\lambda_{max}=4.017,\;C.R.=0.010<0.1⟹Accept$							

**Table 9:** The top two four-color combinations and their calculation results.

Combinations	Four Colors				Order (*O*)	Complexity (*C*)	Aesthetic Measurement (*M*)
	Color 1	Color 2	Color 3	Color 4			
Combination_1	5.6B 8.0/4.6	2.2YR 4.2/9.0	9.9B 1.4/3.1	2.7Y 0.5/0.7	33.7	22	1.532
Combination_2	0.2Y 7.7/9.4	2.2YR 4.2/9.0	2.7Y 0.5/0.7	9.9B 1.4/3.1	33.3	22	1.514
Combination_3	9.5B 5.5/8.8	8.2BG 8.3/1.1	4.5R 1.5/1.7	5.2BG 0.5/1.4	33.25	22	1.511
Combination_4	9.5B 5.5/8.8	8.2BG 8.3/1.1	6.0PB 2.4/2.6	4.5R 1.5/1.7	32.8	22	1.493

**Table 10:** Statistical results of color-matching aesthetic measurement of healing alternatives.

Alternatives	*M_P_* (Observation Angle_1)	*M_P_* (Observation Angle_2)	*M_P_* (Total)
Alternative_1	0.148	0.162	0.310
Alternative_2	0.476	0.529	1.004
Alternative_3	0.596	0.631	1.227
Alternative_4	0.269	0.264	0.532
Alternative_5	0.400	0.447	0.847
Alternative_6	0.363	0.423	0.787
Alternative_7	0.234	0.243	0.477
Alternative_8	0.271	0.267	0.537
Alternative_9	0.375	0.756 *	1.131
Alternative_10	0.703 *	0.389	1.092
Alternative_11	0.678	0.735	1.413 *
Alternative_12	0.350	0.368	0.719
Alternative_13	0.583	0.646	1.229
Alternative_14	0.590	0.650	1.241
Alternative_15	0.461	0.469	0.931
Alternative_16	0.454	0.465	0.919

“*” represents the maximum value.

**Table 11:** Statistical results of color-matching aesthetic measurement of cyberpunk alternatives.

Alternatives	*M_P_* (Observation Angle_1)	*M_P_* (Observation Angle_2)	*M_P_* (Total)
Alternative_1	0.230	0.232	0.462
Alternative_2	0.234	0.237	0.471
Alternative_3	0.156	0.155	0.311
Alternative_4	0.266	0.260	0.526
Alternative_5	0.153	0.155	0.309
Alternative_6	0.149	0.150	0.299
Alternative_7	0.271	0.269	0.543
Alternative_8	0.164	0.163	0.327
Alternative_9	0.282	0.187	0.469
Alternative_10	0.281	0.286	0.567
Alternative_11	0.212	0.215	0.428
Alternative_12	0.213	0.216	0.429
Alternative_13	0.242	0.238	0.480
Alternative_14	0.336	0.329 *	0.665
Alternative_15	0.428 *	0.328	0.756 *
Alternative_16	0.241	0.238	0.479

“*” represents the maximum value.

**Table 12:** Perceptual evaluation results for healing alternatives.

Alternatives	Score (Observation Angle_1)	Score (Observation Angle_2)	Score (Total)
Alternative_1	0.68	0.69	1.37
Alternative_2	0.80	0.82	1.62
Alternative_3	0.79	0.78	1.57
Alternative_4	0.70	0.75	1.45
Alternative_5	0.78	0.79	1.57
Alternative_6	0.72	0.70	1.42
Alternative_7	0.74	0.76	1.50
Alternative_8	0.79	0.77	1.56
Alternative_9	0.75	0.81	1.56
Alternative_10	0.84 *	0.76	1.60
Alternative_11	0.80	0.82	1.62
Alternative_12	0.73	0.75	1.48
Alternative_13	0.76	0.79	1.55
Alternative_14	0.81	0.83 *	1.64 *
Alternative_15	0.74	0.73	1.47
Alternative_16	0.72	0.75	1.47

“*” represents the maximum value.

**Table 13:** Perceptual evaluation results for cyberpunk alternatives.

Alternatives	Score (Observation Angle_1)	Score (Observation Angle_2)	Score (Total)
Alternative_1	0.80	0.81	1.61
Alternative_2	0.79	0.74	1.53
Alternative_3	0.69	0.68	1.37
Alternative_4	0.84	0.83	1.67
Alternative_5	0.70	0.71	1.41
Alternative_6	0.77	0.75	1.52
Alternative_7	0.83	0.81	1.64
Alternative_8	0.71	0.72	1.43
Alternative_9	0.75	0.69	1.44
Alternative_10	0.79	0.79	1.58
Alternative_11	0.77	0.75	1.52
Alternative_12	0.78	0.76	1.54
Alternative_13	0.80	0.78	1.58
Alternative_14	0.80	0.81	1.61
Alternative_15	0.85 *	0.84 *	1.69 *
Alternative_16	0.77	0.76	1.53

“*” represents the maximum value.

**Table 14:** Analysis results of Pearson correlation coefficient.

	Healing Alternatives	Cyberpunk Alternatives
Correlation coefficient	0.726 **	0.80 1 **

“**” indicates *p* < 0.01.

## Data Availability

The research data supporting this publication are provided within this paper.
